# The Accuracy of Broselow Tape Weight Estimate among Pediatric Population

**DOI:** 10.1155/2016/7042947

**Published:** 2016-09-07

**Authors:** Turki M. AlHarbi, Abdullaziz AlGarni, Fasial AlGamdi, Mona Jawish, Tariq Ahmad Wani, Amani K. Abu-Shaheen

**Affiliations:** ^1^Pediatric Emergency Department, King Fahad Medical City, Riyadh, Saudi Arabia; ^2^Research Center, King Fahad Medical City, Riyadh, Saudi Arabia

## Abstract

*Objective.* To determine the accuracy of the Broselow Tape (BT) versions 2007 and 2011 in estimating weight among pediatric population.* Methods.* A cross-sectional study was conducted at King Fahad Medical City and six schools across Riyadh province on 1–143-month-old children. BT 2007 and 2011 estimated weights were recorded. Both tapes via the child's height produce an estimated weight, which was compared with the actual weight.* Results.* A total of 3537 children were recruited. The height (cm) of the subjects was 97.7 ± 24.1 and the actual weight (kg) was 16.07 ± 8.9, whereas the estimated weight determined by BT 2007 was 15.87 ± 7.56 and by BT 2011 was 16.38 ± 7.95. Across all the five age groups, correlation between actual weight and BT 2007 ranged between 0.702 and 0.788, while correlation between actual weight and BT 2011 ranged between 0.698 and 0.788. Correlation between BT 2007 and BT 2011 across all the five age groups ranged from 0.979 to 0.989. Accuracy of both the tape versions was adversely affected when age was >95 months and body weight was >26 kilograms.* Conclusions.* Our study showed that BT 2007 and 2011 provided accurate estimation of the body weight based on measured body height. However, 2011 version provided more precise estimate for weight.

## 1. Introduction

The care and resuscitation of critically ill and injured children require a thorough and meticulous approach. Drug doses, equipment sizes, and intervention decisions in pediatric emergencies are often based on estimated body weights [1, 2]. Imprecise weight estimations may lead to treatment failure [3]. As an actual measurement of weight is impractical during resuscitation, the development of alternative methods of weight measurement is needed. A variety of methods has been developed over years to estimate rapidly a child's weight. One of these methods used to estimate the weight of pediatric population is the Broselow Tape (BT) [4]. This measuring tool divides children into weight categories based on length.

A review of the literature showed contradictory data related to the accuracy of length-based measurements which may depend on the obesity rate of the population studied. A study conducted in 2002 in Victoria, Australia, concluded that length-based estimations were superior when compared with other calculation aids [5]. Similarly, a study conducted by Varghese et al. on Indian children showed that the BT was the most accurate method of weight estimation in children less than 1 year of age [6]. On the contrary, Ken Milne et al. reported that although the BT remains an effective method for estimating pediatric weight, it was not accurate and tended to underestimate the weight of Ontario children [7].

A recent report suggests that length-to-weight estimate may be significantly underestimating weight in USA children due to rising childhood obesity [8]. In view of this fact about childhood obesity, a length-to-weight estimate system such as the BT should be reassessed. In Saudi Arabia, childhood obesity has increased at an alarming rate [9, 10]. The studies conducted during 1988 and 2005 on Saudi school boys from Riyadh stated evidence on the rising trends in body mass index, body fat percentage, and central adiposity that develop obesity [11]. Studies all across Switzerland [7], Australia [5], and South Korea [12] have been performed for validating the BT. However, only a few studies are available from developing countries, including those in the Middle East [13]. Saudi Arabia, like many other countries in the Middle East, has no published data for validation of BT among pediatric population. Moreover, this study is one of the few studies evaluating the accuracy of BT 2011. In light of increasing childhood obesity and the conflicting results of several studies to assess the accuracy of the BT, we aimed to conduct this study to determine the accuracy of the BT versions 2007 and 2011 in estimating weight among Saudi pediatric population.

## 2. Materials and Methods

### 2.1. Study Design

A cross-sectional study was conducted between September 2012 and May 2013 to assess the accuracy of the BT versions 2007 and 2011 in estimating weight among pediatric population in Saudi Arabia.

### 2.2. Setting and Participants

The study was performed in pediatric emergency and outpatient department outpatient clinicss at King Fahad Medical City (KFMC) and six elementary schools (governmental and private) across Al Riyadh province, Saudi Arabia.

Inclusion criteria were children aged from 1 month to 143 months. Exclusion criteria were children needing resuscitation, children presented with severe dehydration, joint contractures, and chronic or endocrine diseases, children with length <46 or >143 cm (prerequisite for using the BT), and children whose parents refused to participate.

### 2.3. Study Protocol

Measurements were taken by five certified nurses. In addition, to standardize the performance of the data collectors, several training sessions on height and weight measurement techniques were provided with the use of same scales that were used in the study to measure the height and weight.

In the pediatric emergency and outpatient departments, children younger than 1 year were weighed wearing a diaper only using Tronix Scale 4800 (accuracy, 0.003 kg; New York, NY). Children 1 year of age or older were weighed wearing ambient clothing using Tronix Scale (accuracy, 0.100 kg).

The height of the children younger than 1 year or those unable to stand was measured in the supine position using Infantometer (accuracy, 3 mm; Perspective Enterprise, Portage, MI). Otherwise, standing length was measured by Stadiometer (accuracy, 0.375 cm; Harpenden Stadiometer; Holtain, Crymych, Great Britain).

In the schools, all measurements were performed with shoes removed and with children wearing light clothing. Standing length (cm) and weight (kg) were measured up to the first decimal place. Body weight was measured by Detecto medical scale (Detecto Scales, Webb City, MO) (accuracy, 0.25 kg). All scales were calibrated by a biomedical engineer at KFMC according to manufacturer's instructions. BT 2007 and 2011 estimated weights were subsequently recorded. Both tapes via the child's height will produce an estimated weight, which can be directly compared with the patient's actual weight.

Primary outcome measure was to check the accuracy of the 2007 and 2011 BT and thereby predict an accurate estimate of a child's weight within a certain agreement. The actual magnitude of the difference was determined as the difference between actual weight and estimated tape weight, with no regard to the sign.

Distribution of these differences and anthropometric measurements were represented by descriptive statistics measures.

Age was grouped into five classes: 1 to 11, 12 to 35, 36 to 59, 60 to 95, and 96 to 143 months. Actual weight was classified as less than 10, 10–20, 20–30, and more than 30 kilograms.

### 2.4. Ethical Consideration

Written informed consent was obtained from the parents/guardians of all children. This study also was approved by the Research Ethics Committee at KFMC, Riyadh, Saudi Arabia, and the administration of each participating school. IRB number was 12-079.

### 2.5. Statistical Analysis

Data was described as mean ± SD (minimum and maximum value), median, and percentages. Chi-square test predicted the association between categorical variables. Paired *t*-test was used to measure the difference between actual weight and tape estimate. Linear regression models postulated difference in tape estimate from actual weight as a function of age. Intrascale correlation coefficients (ICC), Cronbach's Alpha, and Bland-Altman limits were used to determine internal consistency and reliability of agreements.

Reliability was measured by two different examiners measuring a subset of the children (*n* = 578) twice. Cronbach's Alpha and ICC determined the internal consistency of examiners. Statistical tests were performed using SPSS, version 22 (SPSS Inc., Chicago, IL, USA).

## 3. Results

Three thousand five hundred thirty-seven children were assessed, and age ranged from 1 month to 143 months, with a mean age (months) of 48.1 ± 37.9, median age was 37 [1–143], and 59.3% were boys. We combined the results for boys and girls because separate gender analysis showed similar conclusions. The height (cm) of the subjects was 97.7 ± 24.1 [46–143], and the actual weight (kg) of the studied subjects was 16.07 ± 8.9 [1.7–79.2], whereas the estimated weight determined by BT 2007 was 15.87 ± 7.56 and by BT 2011 was 16.38 ± 7.95. The mean difference between actual weight and BT 2007 or BT 2011 was independently significant (*P* < 0.001). Moreover, BT 2007 underestimated weight compared to BT 2011, which tended to overestimate weight (Table 1).

Absolute magnitude of mean difference between actual weight and BT 2007 or BT 2011 had significantly (*P* < 0.001) varied across the subsequent age groups between 1.1 and 4.6 (Table 2). BMI was considerably more in 96- to 143-month age group which did differ significantly (*P* < 0.001).

Table 3 displays the mean differences between the Broselow-predicted weights 2007 and 2011 and actual weight and the internal consistency for BT 2007 and 2011 according to different age groups.

Among 1- to 11-month-old children, BT 2011 was similar to 2007 version in such an agreement (ICC was 0.987). Moreover, the internal consistency for Tape 2007 was 0.817 and for Tape 2011 was 0.821. The mean difference between actual weight and BT 2007 or 2011 was not significant ((*P* = 0.096) and (*P* = 0.740)), respectively. In 12- to 35-month-old children, the internal consistency for Tape 2007 was 0.880 and for Tape 2011 was 0.881, which are considered good estimates.

Moreover, the mean for actual weight was 11.09 ± 2.51; BT 2007 was 11.44 ± 2.54 and BT 2011 was 11.67 ± 2.69. The mean difference between actual weight and BT 2007 or BT 2011 was significant ((*P* < 0.001) and (*P* < 0.001)), respectively, while among 36- to 59-month-olds, the agreement between BT 2011 and 2007 versions was ICC = 0.983; moreover, the agreement between actual weight and BT 2007 was similar to the agreement between the actual weight and BT 2011, ICC = 0.711, 0.716, respectively.

The internal consistency for Tape 2007 was 0.827 and for Tape 2011 was 0.822, which are considered good estimates. The intragroup variance between actual weight and BT 2007 or 2011 was significant ((*P* < 0.001) and (*P* < 0.001)), respectively. The internal consistency for Tape 2007 was 0.800 and for Tape 2011 was 0.790 among 60- to 95-month-old children, which are considered good estimates. The mean of actual weight was 20.43 ± 5.23; BT 2007 was 20.52 ± 3.56 and BT 2011 was 21.10 ± 3.74. The mean difference between actual weight and BT 2007 was not significant (*P* = 0.530), while the mean difference between actual weight and BT 2011 was significant (*P* < 0.001). Among 96- to 143-month-old children the agreement between BT 2011 and 2007 versions was ICC = 0.984; moreover, the agreement between actual weight and BT 2007 was almost similar to the agreement between the actual weight and BT 2011, ICC = 0.702, 0.701, respectively.

Furthermore, the internal consistency for Tape 2007 was 0.751 and for Tape 2011 was 0.728, which are considered good estimates. Mean for actual weight was 29.19 ± 8.96 [6.2–79.2]; BT 2007 was 27.13 ± 4.62 [7–34] and BT 2011 was 28.31 ± 5.06 [7–36]. The mean differences of actual weight and both versions of BT were significant ((*P* < 0.001) and (*P* < 0.001)), respectively.

Reliability analysis for intraclass correlation coefficient with 95% CI about absolute agreement of BT estimates versions 2007 and 2011 was 0.915 [0.842, 0.955] and 0.916 [0.845, 0.956], respectively. The reliability of estimated BT 2007 and 2011 measurement against the actual body weight was estimated by Cronbach's Alpha; both version BT 2007 and version BT 2011 attained same score of (0.95) and (0.94) which affirmed high level of agreement and consistency in the measurements (Table 4).

**Figure 1:**
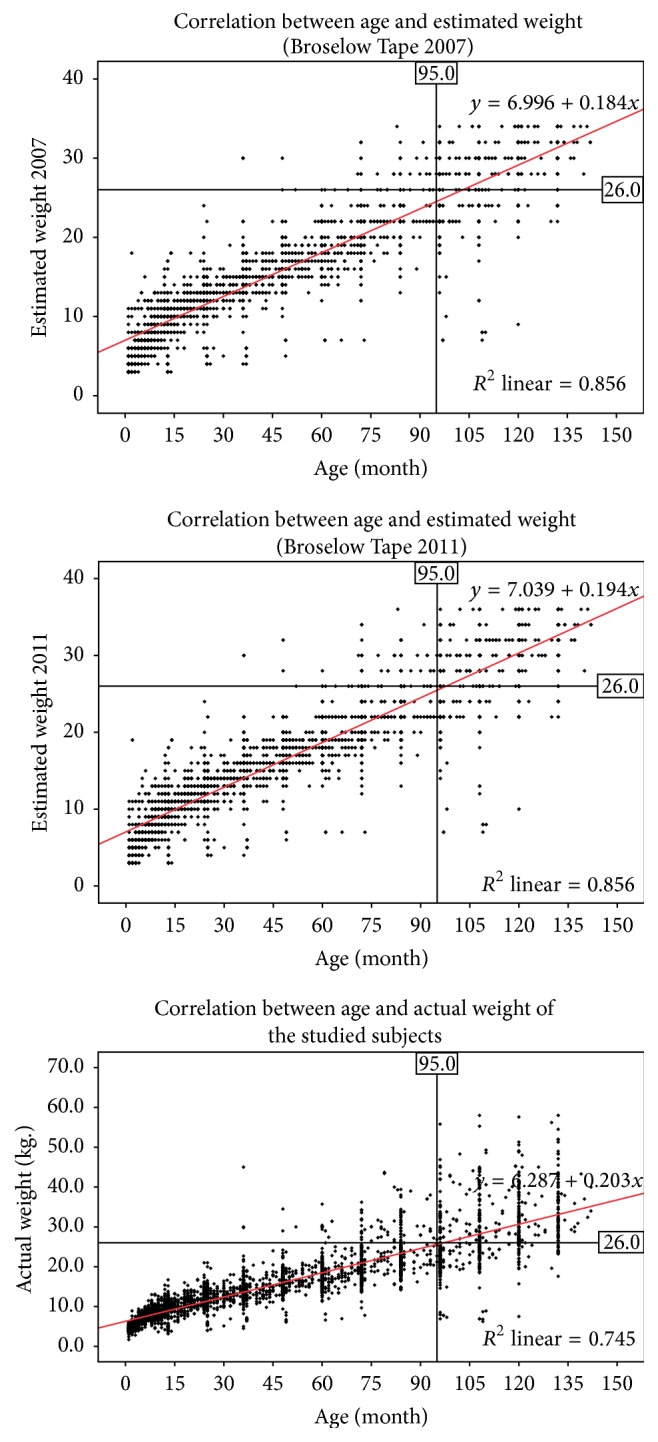
Correlation between age, actual weight, and estimated weight (BT 2007 and 2011).

Mean sign difference (95% CI) for 2007 and 2011 Tape was 0.2 [0.1–0.3] and −0.3 [−0.4–−0.2], respectively. However, mean absolute difference (magnitude with no regard to the sign) was 2.1 [2.0–2.2] and 2.2 [2.1–2.3], thereby indicating similarity in accuracy for both Tape versions (Figure 1).

**Figure 2:**
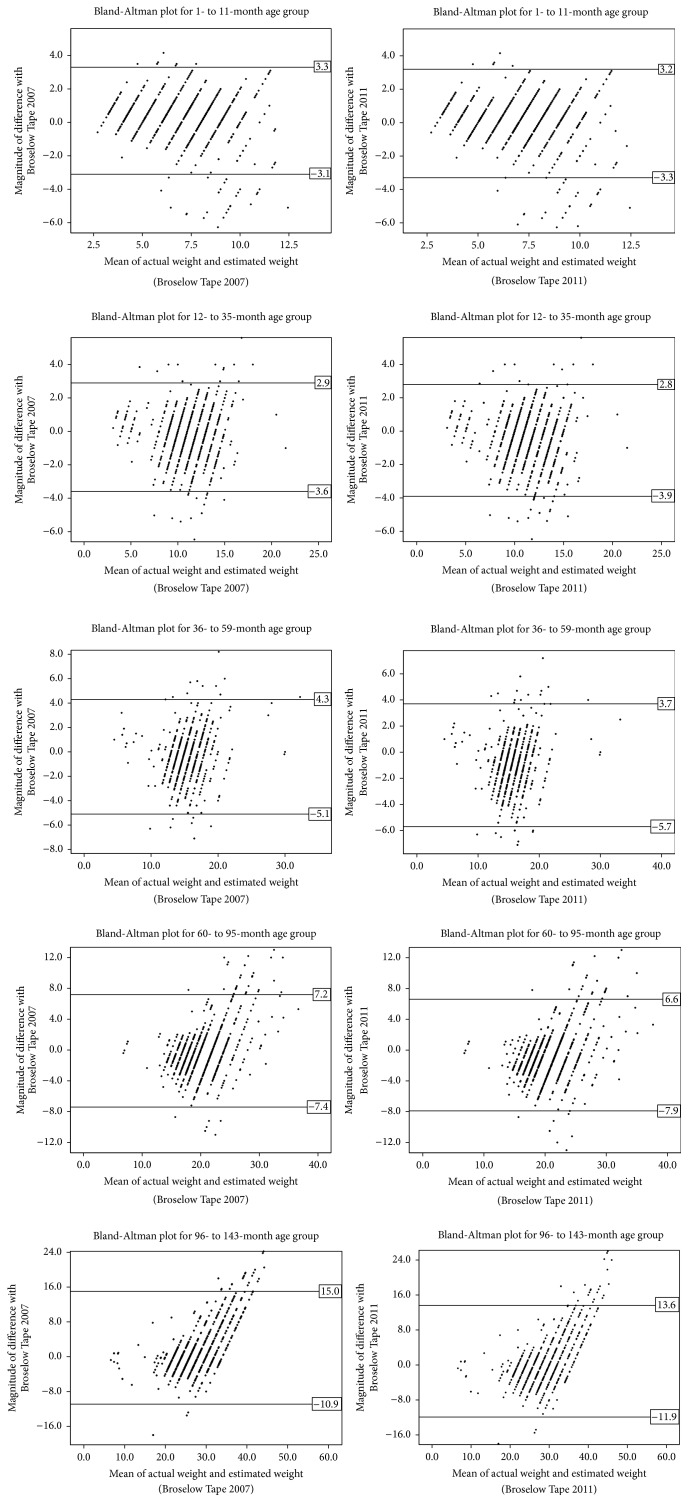
Bland-Altman plot of the mean differences between the actual weight and estimated weight (BT 2007 and 2011) for different age groups.

Across all the five age groups, correlation between actual weight and BT 2007 ranged between 0.702 and 0.788, while correlation between actual weight and BT 2011 ranged between 0.698 and 0.788. Correlation between BT 2007 and BT 2011 across all the five age groups ranged from 0.979 to 0.989. The meager variation between BT 2007 and BT 2011 across all the five age groups was statistically significant which is evidenced by the overestimated results of BT 2011 and large sample size. Accuracy of both tape versions was adversely affected when age was > 95 months and body weight was > 26 kilograms (Figure 2). Bland-Altman trend analysis did clarify that the magnitude of difference for actual weight with BT 2007 or BT 2011 had considerably increased with age.

## 4. Discussion

The BT was developed on the basis of US population data. We conducted this study to determine the accuracy of the BT versions 2007 and 2011 in estimating weight among Saudi children. World Health Organization's 2006 growth standards suggest that genetic influences on childhood weight for length are minimal and, indeed, that breastfed children growing around the world in healthy communities are expected to attain similar heights and weights for given ages [14]. Reports from developing countries show disparity in the prevalence of overweight and obesity. A high prevalence similar to that in developed countries has been reported from countries like Qatar, United Arab Emirates, and Kuwait [15–17]. Al Herbish et al. have studied the Saudi children body mass index and illustrated similarities among the Saudi 282 children and western children [18]. This hypothesis indicates that the same anthropometric methods used to predict children's weights in the United States would be useful in similar settings in KSA. High socioeconomic status against other Asian nations and current life style (energy imbalance as a result of minimal outdoor activity and changes in dietary habits in KSA) has highly influenced such an increase in body weight. Similar to studies done in western populations and Korea [12], our study showed that BT 2007 and 2011 were accurate in estimating the weight, whereas, various studies in a number of different populations, including those done in Canada, have shown that the BT is not an accurate tool in estimating the body weight of pediatrics based on measured body height [7, 19]. The 2011 version of the BT may perform better as it incorporates revised length weight zones based on the most recent National Health and Nutrition Examination Survey (NHANES) data set. However, there was a tendency for loss of accuracy of the Broselow-predicted weights as the child's weight increased, especially when the age exceeds 95 months and the weight is more than 26 kg. Our study raises important questions about the internationalization and validation of the BT for pediatric population in developing countries. Consequently, we demonstrated that BT could be safely used in younger Saudi pediatric population but there is the potential for improvement to modify the tape to suite different populations and age groups. This result was similar to that of the earlier study that reported that the BT accurately measures pediatric emergency needs of Indian children younger than 72 months of age[6]. Theron et al. also showed that the BT underestimated the weights of Pacific Island and Maori children younger than 120 months of age [20]. Furthermore, a similar significantly decreasing accuracy trend has been previously documented using the BT in children weighing more than 25 kg [21]. Another study has shown BT to be accurate in all weights especially in those under 10 kg [22].

The accuracy of the tape was also stratified by gender and found to be similar for the 2007 and 2011 versions.

The strength of our study is that the data were collected from tertiary referral center and elementary schools (governmental and private), distributed across Al Riyadh province, Saudi Arabia. Therefore, it is acceptable to assume that the enrolled children of this study were representative of the Saudi pediatric population. In addition, this study is one of the few studies that evaluate the accuracy of both BT 2007 and BT 2011. Moreover, a subset of children was chosen to evaluate intra- and interexaminers reliability. Each examiner measured the same child twice in this subset. The major strength predicted in this study was the high level of agreement and consistency within and between examiners, while the potential limitation in this study is that the study was conducted on children ≤ 12 years old.

The BT has been found to be the most accurate method when compared with other available methods of weight estimation. Our study adds to the current literature in demonstrating that BT 2007 and 2011 provided accurate estimation of the body weight based on measured body height. However, 2011 version gave a more precise estimate of weight. The accuracy of both BT versions was adversely affected when age exceeded 95 months and weight was more than 26 kg. Health care professionals should consider this information when using the BT to estimate weight for pediatric resuscitation.

## Figures and Tables

**Table 1:** Accuracy of BT for estimation of body weight among participants.

Body weight	Mean ± SD	Absolute mean difference	Correlation	Cronbach's Alpha	*P* value
Actual weight (kg)	16.07 ± 8.93	0.198^a^	0.915^a^	0.998	<0.001^a^
BT					
2007	15.87 ± 7.56	0.308^b^	0.997^b^	0.953	<0.001^b^
2011	16.38 ± 7.95	0.506^c^	0.917^c^	0.949	<0.001^c^

Note: the three paired comparisons between actual weight and BT 2007, actual weight and BT 2011, and BT 2007 and BT 2011 are represented by superscript “a, b, and c.”

**Table 2:** Body mass index (kg/m^2^) and absolute magnitude of mean difference.

Age (month)	BMI (kg/m^2^)	Absolute magnitude of mean difference with BT 2007	Absolute magnitude of mean difference with BT 2011
	Mean ± SD	Mean	Mean
1–11	16.4 ± 3.0	1.1	1.1
12–35	15.5 ± 2.4	1.2	1.3
36–59	14.8 ± 2.4	1.7	1.8
60–95	15.3 ± 2.7	2.6	2.7
96–143	17.1 ± 3.9	4.6	4.6

**Table 3:** Actual weight measurement in comparison with BT measurements according to different age groups.

Age (month)	Body weight	Mean ± SD	Median [min–max]	Correlation	Cronbach's Alpha	*P* value
1–11	Actual weight (kg)	7.17 ± 1.94	7.2 [1.7–13.1]	0.705^a^	0.993	0.096^a^
	BT					
	2007	7.07 ± 2.26	7 [3.0–18.0]	0.987^b^	0.817	<0.001^b^
	2011	7.19 ± 2.25	7 [3.0–19.0]	0.698^c^	0.821	0.740^c^

12–35	Actual weight (kg)	11.09 ± 2.51	11 [3.2–21.0]	0.788^a^	0.993	<0.001^a^
	BT					
	2007	11.44 ± 2.54	12 [3.0–24.0]	0.989^b^	0.880	<0.001^b^
	2011	11.67 ± 2.69	12 [3.0–24.0]	0.788^c^	0.881	<0.001^c^

36–59	Actual weight (kg)	15.20 ± 3.36	15 [4.5–45.0]	0.711^a^	0.991	<0.001^a^
	BT					
	2007	15.62 ± 2.75	15 [4.0–31.0]	0.983^b^	0.827	<0.001^b^
	2011	16.20 ± 2.81	16 [4.0–32.0]	0.716^c^	0.822	<0.001^c^

60–95	Actual weight (kg)	20.43 ± 5.23	19.7 [6.6–43.7]	0.702^a^	0.989	0.530^a^
	BT					
	2007	20.52 ± 3.56	20 [7.0–34.0]	0.979^b^	0.800	<0.001^b^
	2011	21.10 ± 3.74	20 [7.0–36.0]	0.705^c^	0.790	<0.001^c^

96–143	Actual weight (kg)	29.19 ± 8.96	27.4 [6.2–79.2]	0.702^a^	0.990	<0.001^a^
	BT					
	2007	27.13 ± 4.62	28 [7.0–34.0]	0.984^b^	0.751	<0.001^b^
	2011	28.31 ± 5.06	28 [7–36]	0.701^c^	0.728	<0.001^c^

Note: superscripts “a, b, and c” represent comparison between actual weight and BT 2007, BT 2007, and BT 2011 and actual weight and BT 2011.

**Table 4:** Examiners reliability and correlation between actual weight and BT estimates.

	Actual weight	Broselow Tape 2007	Broselow Tape 2011	95% CI on ICC	Cronbach Alpha
Actual weight	1.0000	0.9150	0.9167		0.9978
Estimated weight 2007	0.9150	1.0000	0.9969	0.9755 to 0.9782	0.9532
Estimated weight 2011	0.9167	0.9969	1.0000	0.9768 to 0.9795	0.9487
